# A Weight‐Inclusive Approach to Restrictive Eating Disorders: De‐Centering Weight

**DOI:** 10.1002/eat.24583

**Published:** 2025-10-20

**Authors:** Sarah Nutter, Sage DaSilva, Jessica F. Saunders

**Affiliations:** ^1^ Faculty of Health University of Victoria Victoria British Columbia Canada; ^2^ Brigham and Women's Hospital Boston Massachusetts USA

**Keywords:** anorexia nervosa, atypical anorexia nervosa, weight stigma

## Abstract

**Objective:**

To examine the case for de‐centering weight in the diagnosis of both anorexia nervosa (AN) and atypical anorexia nervosa (AAN).

**Method:**

We summarized research examining the weight‐based similarities and differences between AN and AAN as well as how weight is measured and discussed in research on AAN.

**Results:**

We suggest that weight‐based differences in the diagnosis of AN and AAN may unintentionally perpetuate weight stigma experienced by individuals with AAN throughout the diagnostic and treatment processes. We extend the work of previous researchers by considering how AAN and AN might be considered as one disorder that occurs across the weight spectrum, with presentation and severity specifiers that remove a focus on BMI and enable more equitable access to care for those with AAN.

**Discussion:**

We propose further examination and scholarly discussion for the conceptualization of AN and AAN as one disorder that occurs across the weight spectrum.

**Summary**

- The similarities and differences between anorexia nervosa (AN) and atypical anorexia nervosa (AAN) have received increased research attention in recent years.
- Much of the research examining these similarities and differences has focused on weight‐based differences.
- We propose the conceptualization of AN and AAN as one disorder characterized by restriction and occurring across the weight spectrum.

## 1 Introduction

Increased scholarly attention given to Atypical Anorexia Nervosa (AAN) has contributed to important research examining symptom presentation and severity, diagnostic prevalence, and treatment outcomes (Johnson‐Munguia et al. 2024; Walsh et al. 2023). This work has also led to increased scholarly conversations about AAN in comparison to “typical” Anorexia Nervosa (AN; Birgegård et al. 2023; Golden 2023; Himmerich et al. 2024). This dialog includes rich discussion on the definition and diagnosis of AAN, how it differs from AN, and whether those differences are meaningful enough to retain AAN and AN as separate diagnoses.

An important area of research on AAN has been qualitative research documenting the lived experiences of those with AAN in treatment (Harrop et al. 2023, 2025). Individuals with AAN have described weight stigma from healthcare professionals throughout their illness, including: (1) being pathologized for higher weights and encouraged to lose weight; (2) the lack of recognition of eating disorder symptoms and severity of illness; and (3) weight‐centric, rather than weight‐neutral, treatment that is focused on weight and weight loss, often with inadequate nutritional care, insensitive interventions, and under treatment (Harrop et al. 2023, 2025). These findings highlight the ways that weight stigma and fear of fat intentionally and unintentionally impede the treatment of those with AAN. While weight stigma has been acknowledged in discussions about the definition and diagnosis of AAN, we believe there is a need for a deeper examination of how these sociocultural attitudes may be influencing this discussion.

The purpose of this Spotlight is to consider the case for de‐centering weight in the diagnosis and treatment of AAN and AN. We recognize that weight may be an important piece of an individual's overall illness severity and treatment plan, and that it is important to ensure weight‐based conversations in treatment are conducted in a highly sensitive manner (Laboe et al. 2025). However, we also believe it is important for researchers and clinicians to critically examine how sociocultural attitudes about weight, weight gain, and weight loss may be unintentionally influencing our research and clinical practices, and to interrogate how using weight as the differential factor between AAN and AN is inherently weight stigmatizing. Our discussion will first consider the ways that weight is measured and discussed in the AAN literature, followed by a summary of recent work examining the similarities and differences between AAN and AN, and ending with a consideration of recent work on clarifying the diagnosis of AAN, expanded to consider its applicability across the weight spectrum.

## 2 Weight‐Based Investigations of AAN

A major focus of scholarly discussion on AAN has been weight‐based, much of it pertaining to the unclear diagnostic criterion of “significant” weight loss. Researchers have examined different definitions of significant weight loss and its implications for diagnosis (Forney et al. 2017), the relationship between weight, weight loss, and medical instability in patients with AN and AAN (Brennan et al. 2023; Peebles et al. 2010), and how the amount, rate, and duration of weight loss predict illness severity (Garber et al. 2019). Findings have indicated that, regardless of diagnosis, rapid weight loss as opposed to overall weight predicts medical instability (Brennan et al. 2023; Garber et al. 2019). Researchers have also examined how weight influences clinical decision making in eating disorder care contexts, finding that both the percentage of total body weight lost as well as total body weight following weight loss had an impact on clinicians' confidence in an AAN diagnosis (Beard et al. 2025). Symptoms of AN are more likely to result in diagnosis for lower‐weight versus higher‐weight individuals, contributing to missed opportunities for diagnosis (Loeb et al. 2023). Duration of weight loss and lifespan weight fluctuations have also been described as complicating the accurate diagnosis of AAN (Krug et al. 2024).

Researchers investigating differences in treatment outcomes have examined length of time to reach treatment goal weight, noting that those with AAN require less time to reach treatment goal weight and are more likely to reach their goal weight, but are just as likely as patients with AN to require re‐hospitalization (Shachar‐Lavie et al. 2022). In their study, patients with AN and AAN had similar age of onset of symptoms and similar severity of depressive symptoms at intake, but those with AAN presented to treatment at a later age (Shachar‐Lavie et al. 2022), consistent with research documenting barriers to diagnosis and timely treatment for higher‐weight patients with eating disorders (Harrop et al. 2021; Kennedy et al. 2017). Further, treatment goal weight is itself a contested topic in AAN treatment, with the goal at times being weight stabilization, rather than weight restoration (Quon and Kelly 2023), which may reflect the lower length of time to reach goal weight previously identified by Shachar‐Lavie et al. (2022).

In a scoping review on the state of knowledge on AAN, three of the four recommendations for research were weight‐related (Beard and Waller 2024), specifically to: (1) note the current weight of patients; (2) describe how significant weight loss was operationalized; and (3) note whether weight loss is ongoing. Both clinical practice recommendations were focused on weight loss, in an effort to support clinicians in reaching an accurate diagnosis to inform treatment directions (Beard and Waller 2024). In a review of inconsistencies in diagnostic criteria for eating disorders in the other specified feeding and eating disorder category, researchers noted a common misconception that those with AAN are only slightly above the BMI cutoff for AN, despite most individuals with AAN having body weights in the middle and upper weight categories (Krug et al. 2024).

In calling attention to the focus on weight in the AAN literature, it is not our intention to criticize the research of our colleagues or to imply our colleagues have not engaged in similar critical consideration of the complexity of the discussion on weight. Our intention is to simply call attention to how the weight‐based criteria of AN and AAN influence our research, clinical practice, and scholarly discussions on restrictive eating disorders and potentially harm those with higher weight (Harrop et al. 2023, 2025).

## 3 AAN and AN: Similarities and Differences

Existing literature reveals numerous similarities between AN and AAN, emphasizing that a patient's presenting weight is one of the few distinguishing characteristics that enables clinicians to differentiate between the disorders (Billman Miller et al. 2024; Fitterman‐Harris et al. 2024). Clinically indistinct features of AN and AAN include psychiatric comorbidities, psychological and physiological symptoms, and treatment outcomes (Billman Miller et al. 2024; Crumby et al. 2024; Golden and Walsh 2024; Urban et al. 2025). Specifically, research findings indicate that individuals with AAN show levels of ED psychopathology (e.g., restrictive eating, body dissatisfaction, preoccupation with food, shape, and weight) that are greater than or equal to those with AN (Johnson‐Munguia et al. 2024; Walsh et al. 2023). Similarly, the physiological consequences of restrictive eating have been found to impact individuals with AAN at comparable or greater rates than individuals with AN, aside from changes in bone health during and after illness (Vo and Golden 2022; Walsh et al. 2023). Literature on treatment outcomes also closely corresponds, illustrating that individuals with AN and AAN recover from the psychological symptoms of these restrictive disorders at a similar pace (Golden 2023; Urban et al. 2025).

Despite these shared clinical features, the process of diagnosing individuals with AAN does not mirror that of patients with AN. In particular, research suggests individuals with AAN take approximately 10 months longer to receive a diagnosis than patients with AN, often as a result of clinical misdiagnosis or dismissal perpetuated by weight stigma (Kons et al. 2024; Lebow et al. 2015). Findings reported by Cunning and Rancourt (2024) highlight the potential impact of illness stigma and perceptions of controllability on the diagnosis of AAN. Similarly, Veillette et al. (2018) noted that the likelihood of receiving an AN or AAN diagnosis from a mental health provider increased when a patient appeared to be ‘underweight’, highlighting a potential stereotype of restrictive eating disorders as occurring only among thin individuals. These findings reflect widespread uncertainty surrounding the recognition and diagnosis of AAN.

Notably, the belief that patient admission weight is an accurate indicator of ED severity is a common and inaccurate misconception (Garber et al. 2019; Whitelaw et al. 2018). Studies examining the entire weight history of individuals in treatment for restrictive EDs suggest that total weight loss and recent weight loss are more accurate predictors of psychological and physiological ED symptoms than admission weight (Garber et al. 2019; Whitelaw et al. 2018). In other words, this implies that a patient who appears ‘overweight’ despite recently losing weight from extreme food restriction may have a higher risk for ED complications than a visibly ‘underweight’ patient. Moreover, Hebebrand et al. (2024) observed that the premorbid body weight of an individual was a stronger predictor of their total weight loss than whether they were diagnosed with AN or AAN, and suggested that premorbid weight and weight loss may be stronger criteria for treatment admission than current BMI. Taken together, these findings substantiate the claim that there is likely no meaningful distinction between AN and AAN, providing additional support for the call to conceptualize the two diagnoses as a single underlying disorder (Hebebrand et al. 2024).

## 4 Weight Neutral Diagnosis of Restrictive Eating Disorders

In their examination of clinicians' perspectives on the definition and diagnosis of AAN, Beard and Waller (2024) found that 53% of clinicians in their study noted clearer guidance is needed on the weight criterion, 19% indicated a perceived issue with the reference of AN diagnostic criteria in the criterion for AAN, and 27% indicated concerns about whether or not a separate diagnosis of AAN is warranted, given the similarities between AN and AAN and the potential harms of weight stigma. Similarly, Golden (2023) noted that a more inclusive category of restrictive eating disorders, independent of body weight, may enable access to care. We echo these concerns about the potential harms of weight stigma and sociocultural attitudes about weight and agree that a more inclusive diagnosis may be warranted.

Recently, researchers proposed the renaming of AAN to restrictive eating disorder, refining diagnostic criteria via a critical discussion of AAN in relation to the criterion for AN (Birgegård et al. 2023). They asserted that dietary restriction should represent the foundation of the diagnosis, with weight and weight loss being incorporated as presentation specifiers, given both the complexity and lack of diagnostic clarity for the significant weight loss criterion. They noted that fear of weight gain may present differently across the weight spectrum, but that severe and enduring weight‐related distress may provide enhanced diagnostic flexibility. Both criteria of disturbed experience of body shape or weight, as well as undue influence of weight or shape on self‐evaluation, were described as equally suitable for patients with AAN compared to those with AN. Finally, they proposed that the lack of recognition of the seriousness of low weight be rephrased to a lack of recognition of the psychological and physical health consequences of extreme restriction (Birgegård et al. 2023).

While we agree with the critiques and suggestions of Birgegård et al. (2023), we question why these criteria should apply only to AAN. Is restriction not the cornerstone of both AN as well as AAN? Is the lack of patient recognition of the health consequences of restriction not what the lack of recognition of the seriousness of low weight AN criterion implies? If the underlying symptomatology, presentation, treatment approach, and risk of relapse are similar for those with AN and AAN, it seems that expectations for weight, weight loss, and weight gain in treatment are the only meaningful barriers preventing the conceptualization of AAN and AN as one disorder occurring across the weight spectrum. We believe it is important to consider the possibilities for weight, weight loss, and medical complications to, instead, be presentation and severity specifiers that enable clinicians to engage in treatment planning, especially given research findings documenting the impact of weight stigma on the AAN diagnostic and treatment process (Harrop et al. 2023, 2025). One diagnostic category for both AN and AAN would allow for all patients to receive important severity specifiers, for those specifiers to be focused on health markers, regardless of patient weight, and to better account for racial and ethnic differences in weight and body fat distribution (Karnes et al. 2021).

Overall, we believe that simply renaming AAN to restrictive eating disorder while retaining it as a separate diagnosis would retain the issue of a weight‐based hierarchy in perceptions of those with AAN as “not sick enough”, by both patients and providers (Eiring et al. 2021; Harrop et al. 2023, 2025). While additional research is needed to better understand similarities and differences between AN and AAN for genetic risk, response to psychotherapy and medications, as well as long‐term outcomes, we hope our perspectives provide new considerations for the possibility of greater inclusion and recognition of eating disorders as occurring across the weight spectrum.

## 5 Conclusion

Our perspective is consistent with that of Golden (2023) as well as Himmerich et al. (2024), who, in their commentary on the evolution of eating disorder diagnoses, stated that assessment and treatment must be sensitive to the needs of all individuals with eating disorders, including those with atypical presentations. However, we also argue that the categorization of AAN as atypical is weight stigmatizing and heavily influenced by sociocultural attitudes toward weight and appearance. Only by fully interrogating our historical and current reliance on weight in the diagnosis and treatment of AN and AAN, and the biases inherent in this reliance, will we be able to better understand how this will serve (or not serve) research and clinical practice moving forward.

## Author Contributions

**Sarah Nutter:** conceptualization, writing – original draft, writing – review and editing. **Sage DaSilva:** writing – original draft. **Jessica F. Saunders:** conceptualization, writing – review and editing.

## Conflicts of Interest

The authors declare no conflicts of interest.

## Data Availability

Data sharing not applicable to this article as no datasets were generated or analysed during the current study.
